# Valsalva retinopathy in pregnancy: a case report

**DOI:** 10.1186/1752-1947-2-101

**Published:** 2008-04-07

**Authors:** Abdullah S Al-Mujaini, Carolina C Montana

**Affiliations:** 1 Department of Ophthalmology, Sultan Qaboos University Hospital, Alkhod, Muscat, Sultanate of Oman

## Abstract

**Introduction:**

Valsalva retinopathy is a unilateral or bilateral condition that occurs when increased intra-thoracic or intra-abdominal pressure transmitted to the eye causes a sharp rise in the intra-ocular venous pressure, and rupture of superficial retinal capillaries. The patient often gives a history of a recent strenuous physical act, which could have increased the intra-thoracic pressure. Pregnancy is known to be a risk factor for Valsalva retinopathy.

**Case presentation:**

A 23-year-old woman in her seventh month of pregnancy presented with a history of decreased vision in her left eye of one-week duration. Examination of the affected eye showed best corrected visual acuity of 20/50, and fundus examination revealed a pre-retinal hemorrhage located in the macula. Based on clinical findings, the diagnosis of Valsalva retinopathy was made.

**Conclusion:**

Retinal hemorrhages can be generated by Valsalva maneuvers. Pregnancy is a known risk factor for Valsalva retinopathy; however, the diagnosis should be made only after excluding other causes of retinal hemorrhages. It is a self-limited event. We report a case of Valsalva retinopathy complicating normal pregnancy and confirm that, to date, there is no evidence to indicate that there is a risk of recurrence following spontaneous vaginal delivery.

## Introduction

Valsalva retinopathy is a unilateral or bilateral condition that occurs when increased intra-thoracic or intra-abdominal pressure transmitted to the eye causes a sharp rise in the intra-ocular venous pressure, and rupture of superficial retinal capillaries [1]. The patient often gives a history of a recent strenuous physical act, which could have increased the intra-thoracic pressure. Pregnancy is known to be a risk factor for Valsalva retinopathy [2]. We hereby report a case of Valsalva retinopathy in a woman in her seventh month of pregnancy.

## Case presentation

**Figure 1:**
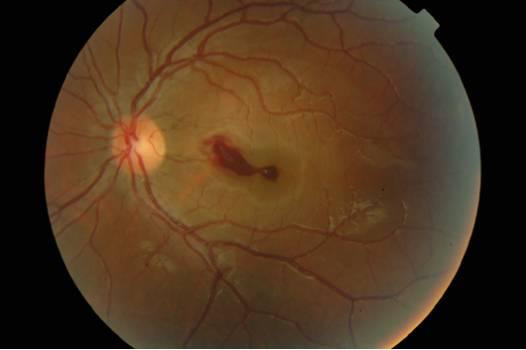
Fundus photograph of the left eye at presentation.

A 23-year-old woman in her seventh month of pregnancy presented with a history of decreased vision in her left eye of one week duration. Her previous medical history was unremarkable but for constipation. She was not on medications. Ophthalmic examination showed best corrected visual acuity of 20/20 in the right eye (OD) and 20/50 in the left eye (OS). The anterior segment was unremarkable and intra-ocular pressure was 10 mm Hg in both eyes. Fundus examination was normal OD, and revealed a pre-retinal hemorrhage located in the macula OS (Figure 1).

**Figure 2:**
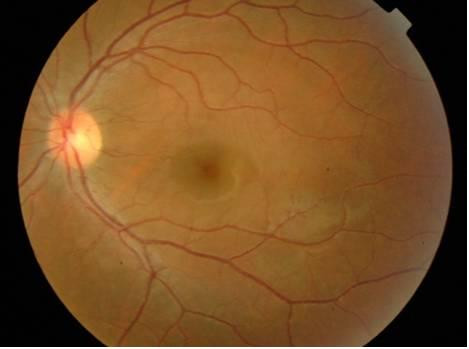
Fundus photograph of the left eye a month later.

Since the patient was pregnant, fluorescein angiography was not performed. Blood pressure, full blood count, coagulation profile, fasting blood sugar and sickle cell tests were within normal limits. Additional tests for a hypercoagulable state and autoimmune diseases were negative. A clinical diagnosis of Valsalva retinopathy was made and it was decided to observe the patient. Follow-up one month later revealed improvement in the best corrected visual acuity to 20/20 OS, with complete resolution of the macular hemorrhage (Figure 2). The patient had a spontaneous vaginal delivery, was seen immediately after the delivery, with no recurrence of the retinal hemorrhage.

## Discussion

Pregnancy exerts multiple hormonal, metabolic, hematological and immunological alterations in the mother that represent risk factors for Valsalva retinopathy. Elevation of intra-abdominal pressure during pregnancy, with a further increase during labor, leads to a considerable elevation in intravenous pressure, which increases the potential for retinal hemorrhages following a Valsalva maneuver. Hematological changes during pregnancy such as thrombocytopenia add to the risk of Valsalva retinopathy in pregnancy.

It is important to rule out all systemic diseases that may result in retinal hemorrhages, such as diabetes, hypertension, sickle cell disease, anemia, coagulopathy, blood dyscrasias and previous ocular vein occlusions.

Whether vaginal delivery poses a risk of recurrence or exacerbation of the hemorrhage is unclear [2]. A review of the literature showed no recurrence of retinopathy following spontaneous vaginal delivery [2,3].

Valsalva maneuvers typically result in sub-internal limiting membrane hemorrhages with a predilection for the macula, but sub-retinal, retinal or intravitreal hemorrhages can occur. The prognosis in general is good and the condition in most patients resolves spontaneously over several months. Some patients may have a poor visual outcome, which has been attributed to retinal pigmentary changes at the macula [3]. In most cases, conservative management is indicated with periodic observation. A YAG laser has been employed in selective cases to disperse the pre-retinal hemorrhages and speed up resolution [4].

## Conclusion

Retinal hemorrhages can be generated by Valsalva maneuvers. Pregnancy is a known risk factor for Valsalva retinopathy; however, the diagnosis should be made only after excluding other causes of retinal hemorrhages. It is a self-limited event. We have reported a case of Valsalva retinopathy complicating normal pregnancy and confirm that, to date, there is no evidence to indicate that there is a risk of recurrence following spontaneous vaginal delivery.

## Competing interests

The author(s) declare that they have no competing interests.

## Authors' contributions

All authors participated in the design of the manuscript. AM was involved in drafting the manuscript for important intellectual content. All authors read and approved the final manuscript.

## Consent

Written informed consent was obtained from the patient for publication of this case report and accompanying images. A copy of the written consent is available for review by the Editor-in-Chief of this journal.
